# High Temperatures and *Bacillus* Inoculation Affect the Diversity of Bradyrhizobia in Cowpea Root Nodules

**DOI:** 10.1002/jobm.70058

**Published:** 2025-05-20

**Authors:** Crislaine Soares Oliveira, Juliane Rafaele Alves Barros, Viviane Siqueira Lima Silva, Paula Rose de Almeida Ribeiro, Francislene Angelotti, Paulo Ivan Fernandes‐Júnior

**Affiliations:** ^1^ Colegiado de Farmácia Universidade Federal do Vale do São Francisco Petrolina Brazil; ^2^ Fundação de Amparo à Pesquisa do Estado de Pernambuco Recife Brazil; ^3^ Embrapa Semiárido Petrolina Brazil; ^4^ Departamento de Tecnologia e Ciências Sociais Universidade do Estado da Bahia Juazeiro Brazil

**Keywords:** *Bacillus* sp, *Bradyrhizobium*, climate change, heat stress, inoculant

## Abstract

Future climatic scenario predictions indicate a substantial temperature increase, reducing crop production worldwide and demanding the development of adaptations in agriculture. This study aimed to assess the impact of high temperatures and amendments with *Bacillus* on nodulating bradyrhizobia. Two cowpea genotypes were evaluated at low (min = 20.0°C, max = 33.0°C) and high‐temperature regimes (min = 24.8 C, max = 37.8°C). Plants were also inoculated with *Bacillus* sp. ESA 402, a plant growth‐promoting bacterium. The molecular diversity of the bradyrhizobia isolated from cowpea nodules and plant growth was assessed. High temperatures reduced nodulation of the BRS Itaim cowpea genotype. One hundred and eighty‐six were genotyped, clustering the collection into 45 groups. The high temperatures reduced the number of groups, but this negative influence was diminished by *Bacillus* inoculation. Alpha diversity showed little impact on the experimental interactions. However, this influence was evident for all factors and the interaction of the three factors when beta diversity was assessed. 16S rRNA and constitutive gene sequences identified all strains as *Bradyrhizobium* spp. mainly within the *B. japonicum* supercluster. Cowpea‐*Bradyrhizobium* association diversity is multifactorial under different temperature regimes, as is the presence or absence of the plant‐growth‐promoting bacteria *Bacillus* sp. ESA 402.

**Abbreviations**
CMISA: Culture Collection of Microorganisms with Agricultural Interest of Embrapa Semiárido (Acronym in Portuguerse)
IPCC: Intergovernmental Panel on Climate Change
PCoA: principal coordinate analysis
PERMANOVA: Permutational multivariate analysis of variance
PGPB: plant‐growth‐promoting bacteria
YEM: yeast‐extract, mannitol
YMA: yeast‐extract, mannitol, agar

## 1 Introduction

Global changes in the Earth's climate affect the physiology and ecology of crops, which will require action to reduce losses in plant growth and yield shortly [1]. Among the agricultural systems affected by climate change, those family‐based systems conducted by smallholders in current semiarid and arid regions will be the most affected worldwide [1, 2]. Adaptation strategies will be imperative to overcome these challenges and reduce climate impacts on smallholder crops. Among these crops, cowpea (*Vigna unguiculata* L. Walp.) deserves attention since it is the primary source of protein and carbohydrates for millions of inhabitants in the South American and African continents [3, 4].

Cowpea is a tropical legume crop moderately tolerant to abiotic stresses (e.g., drought, salinity, and heat), allowing the crop to be grown globally under dryland conditions from the African center of origin to South America [4, 5]. A current reality in the field is boosting cowpea's tolerance to harsh conditions by applying biotechnologies, such as plant breeding to develop more adapted genotypes [3], inoculation with selected rhizobia [6, 7, 8], and plant‐growth‐promoting bacteria (PGPB) [9, 10, 11]. Recent reports have shown that the temperature affects cowpea growth, biochemistry, and reproductive biology [12, 13, 14, 15], so heat tolerance is essential in focusing on plant breeding for future climatic conditions. Exploiting cowpea ecological partners, such as rhizobial microsymbionts and PGPB, could lead to fast cowpea growth and yield improvement under harsh environmental conditions.

Cowpeas nodulate with a wide range of soil alpha‐ and beta‐rhizobia [16]. The main microsymbionts are those *Alphaproteobacteria* belonging to the *Bradyrhizobium* genus. In tropical drylands, the diversity of cowpea‐nodulating strains that form effective nodules in cowpea roots is influenced by several factors, such as soil management [17] and cowpea genotype [6, 18]. Plant‐rhizobia communication is triggered by root exudates (particularly flavonoids) [19], which may be affected by soil conditions, potentially affecting the communication between cowpeas and rhizobia in soil. Environmental factors on cowpea nodulation, such as increasing soil temperatures, have already been investigated [20]. Nevertheless, the influence of air temperature (that impacts plant physiology and, consequently, the root exudation pattern) has yet to be assessed on the cowpea nodulating bacterial community.

Applying PGPB in field and pot experiments has improved cowpea growth and development [9, 10, 11, 21]. However, under high‐temperature conditions, the influences of these inoculants on cowpea development and symbiosis establishment with rhizobia have not been shown. The selection of bacteria to alleviate the impact of abiotic stresses in crops has focused on *Bacillota* (former *Firmicutes*) strains, mainly *Bacillus* spp. [22, 23]. *Bacillus* sp. ESA 402 is a strain isolated from field‐grown sorghum (genotype BRS Ponta Negra) in Petrolina, Pernambuco, Brazil [24]. This bacterium is an efficient sorghum (*Sorghum bicolor*) growth promoter that can induce plant endurance under drought, at least for sorghum [25] and sesame (*Sesamum indicus*) [26, 27] crops, indicating that this bacterium can benefit other plant species, under different abiotic stresses.

Nevertheless, we hypothesized that the increase of 4.8°C in the air temperature, as projected by the Intergovernmental Panel on Climatic Changes (IPCC) report as the worst scenario by the end of the current century [28], and inoculation of an efficient non‐rhizobia PGPB in cowpea genotypes could change the genetic profile of bradyrhizobia in root‐nodules. This study evaluated the growth, nodulation, and bradyrhizobial diversity of two cowpea genotypes subjected to inoculation of *Bacillus* sp. ESA 402 and two temperature regimes.

## 2 Materials and Methods

### 2.1 Plant Materials and Bacterial Strain

Cowpea (*Vigna unguiculata* L. Walp) genotypes BRS Acauã and BRS Itaim were used in the present study. BRS Acauã is a “black‐eyed pea” type cowpea cultivar developed for high yields under rainfed and irrigated systems under the Brazilian drylands conditions. This genotype is also resistant to high temperatures with low flower abortion [3, 29]. In addition, BRS Acauã also associates very well with the native rhizobial community in the Brazilian dryland soils [6, 7]. BRS Itaim is also a “black‐eyed pea” type of cowpea bred for Brazil's semiarid region with high protein, iron, and zinc [30].

The PGPB used in this study was *Bacillus* sp. ESA 402 (16S rRNA GenBank accession number MK424603), a strain belonging to the *Bacillus subtilis* Clade isolated from field‐grown sorghum (*Sorghum bicolor*) in Petrolina, Pernambuco, Brazil. This strain is an auxin producer that can solubilize calcium phosphate and produce siderophores [24]. This strain has already been proven to induce drought‐resistance in crops like sorghum [25] and sesame [26, 27]. This strain is currently deposited in the Culture Collection of Microorganisms with Agricultural Interest (CMISA ‐ Embrapa Semiárido).

### 2.2 Experiment Setup and Cowpea Growth

The experiment was conducted from December 2021 to February 2022 using two growth chambers (Phytotron) with previously set temperature regimes. The first chamber had a minimum temperature of 20.0°C from 8 p.m. to 6 a.m., 26.0°C from 6 to 10 a.m., a maximum temperature of 33.0°C from 10 a.m. to 3 p.m., and 26°C from 3 to 8 p.m. These temperature averages for Petrolina (Pernambuco, Brazil) for the last 30 years. In the second chamber, an increasing temperature of 4.8°C was added to each range (24.8°C from 8 p.m. to 6 a.m., 30.8°C from 6 to 10 a.m., the maximum temperature of 37.8°C from 10 am to 3 p.m., and 30.8°C from 3 to 8 p.m.). We selected the increase of 4.8°C based on the predictions described in the IPCC report as the worst scenario by the end of the current century [28]. The air humidity was constant at 60 ± 5%, and the light intensity was 1500 µmol m^‐2^ s^‐1^ in both chambers.

Cowpea seeds were surface disinfected following Somasegaran and Hoben [31] and sowed in a soil sample (0–0.20 m) collected in the Bebedouro Experimental Field (Embrapa Semiárido, latitude = −9.1373, longitude = −40.3021). The soil, classified as a Loam Ultisol, was cropped with cowpea (non‐inoculated) and maize (*Zea mays*) as previous crops. The soil was sieved (5 mm mesh), homogenized, and used to fill up 500 mL polystyrene pots carefully accommodated on tables in each chamber. The soil sample was chemically analyzed following Teixeira et al. [32] (Table S1), and no fertilization was carried out.

ESA 402 inoculant was prepared by growing the bacteria in a liquid YEM (yeast extract and mannitol) medium for 48 h at room temperature with a constant stirring of 120 rpm. Afterward, the bacterial broth was centrifuged at 6000 *g* for 3 min, and the pellet was resuspended in NaCl (0.85% w v^‐1^), adjusting the optical density to 0.6 at 600 nm spectrophotometrically (OD_600nm_ = 0.6). This process was repeated twice. The seeds inoculated with ESA 402 received 1 mL of the adjusted culture broth on each seed. Treatments without ESA 402 received 1 mL of NaCl (0.85% w v^‐1^).

Three seeds were sown per pot, and 15 days after sowing, the spare plants were thinned out, leaving one plant per pot. The pots were watered as needed, and the plants were harvested 35 days after emergence. Roots, nodules, and shoots were separated, and the nodules were counted before storage at −80°C in pure glycerol until isolation. Roots and shoots were placed in paper bags and dried at 65°C for 5 days for weighing.

### 2.3 Bradyrhizobia Isolation, Molecular Fingerprinting, and Clustering

We randomly selected 10 nodules from each root for isolation in the YMA medium [33]. The nodules were surface disinfected with 95% (v v^‐1^) ethanol for 30 s and NaClO 2% (v v^‐1^) for 5 min, followed by 10 washes in autoclaved dH_2_O [31]. The nodules were crushed and streaked onto YMA plates. The water from the final rinse was streaked onto the YMA medium to confirm disinfection. The plates were incubated for 7 days at 28°C, and the cultures were restreaked in the same medium and reincubated. The pure cultures were obtained after several rounds of purification on YMA medium when preserved at −80°C in glycerol stocks in CMISA.

Two hundred thirty bacteria (out of 240) showed typical bradyrhizobial colonies (growth after 5 days of incubation, white colony color, and alkaline reaction in the YMA medium), and were selected for further analyzes. Selected strains were grown in the YM medium for 6 days, and the DNA was extracted using the commercial kit “Brasílica” (LGC Biotecnologia, São Paulo, Brazil). The bacteria were molecularly authenticated by *nifH* and *nodC* duplex PCR reactions [34]. PCR products were subjected to horizontal electrophoresis in 1% (w v^‐1^) agarose gel with Diamond^TM^ Nucleic Acid Dye (Promega, Fitchburg, USA), and the clear bands of both amplicons indicated the presence of both genes. All *nifH* and *nodC*‐positive strains (186 bacteria) were selected for molecular fingerprinting.

We used the BOX‐PCR molecular fingerprinting approach to establish the profile of the potential different *Bradyrhizobium* in the culture collection. The bacterial fingerprinting was assessed by PCR using the primer BOX A1R (CTACGGCAAGGCGACGCTGACG) [35]. PCR products were subjected to horizontal electrophoresis using 1.25% (w v^‐1^) agarose gel at 90 V for 3 h. All the BOX‐PCR profiles were clustered in a similarity dendrogram using the software BioNumerics v 7.6 (Applied Maths, Belgium), applying the UPGMA method and the Dice coefficient.

### 2.4 Sequencing of the 16S rRNA and Housekeeping Genes

According to the BOX‐PCR profiling, 45 clusters were formed, and one strain from each cluster was selected for identification through 16S rRNA and housekeeping genes sequence analysis (*recA*, *gyrB*, and *rpoB*). PCR amplifications were performed in a ProFlex PCR System (Applied Biosystems). The primers, temperatures, and cycling conditions are listed in Table S2. The PCR products were purified using the EasyPure PCR Purification kit (TransGen Biotech, China). The DNA was Sanger sequenced in Clinilab LTDA (Salvador, Brazil) in an ABI 3500 genetic analyzer (Life Technologies, USA).

The 16S rRNA, *recA*, *gyrB*, and *rpoB* sequences were analyzed using Sequence Scanner 2.0 (Applied Biosystems) for quality control. Good‐quality sequences (QV > 20) were used for comparison analysis against the type strains available in the GenBank database using the BLASTn tool [36]. The sequences of the bacteria isolated in the present study and those from 30 *Bradyrhizobium* reference strains were aligned using the MUSCLE tool in MEGA 11 software [37]. The phylogenetic tree was constructed using the maximum likelihood (ML) method and the Jukes‐Cantor model using the bootstrap statistical method with 1000 replications.

All the sequences are deposited in the GenBank database (https://www.ncbi.nlm.nih.gov/genbank/) with the following accession numbers for the 16S rRNA: PQ490629‐PQ490673; *recA*: PP842410‐PP842456; *gyrB*: PQ480083‐PQ480127; and *rpoB*: PQ480128‐PQ480173.

### 2.5 Statistical Analysis

All statistical analyses were conducted in the R environment v. 4.3.2 using the interface RStudio v. 2023.12.1 + 402 [38]. The plant growth and nodulation data from the growth chambers experiment were analyzed by one‐way analysis of variance (ANOVA) after the Shapiro‐Wilk test assessed the normal distribution of the residuals (errors) and the Levene test assessed variance homogeneity. To assure these assumptions, data for the number of nodules per plant and the number of BOX‐PCR clusters were subjected to Box‐Cox transformation. Following the ANOVA, a two‐tailed t‐test compared significant factors and interactions. These analyses used the *lme4* R package [39].

The similarity dendrogram constructed by analyzing the BOX‐PCR profiles of the 186 bacterial strains was used to build a sheet with the abundance of each bacterial cluster (100% similarity) per pot (sample). This abundance sheet was used to proceed with all analyses of bacterial diversity. The alpha‐diversity indexes (Shannon‐Wiener and Simpson) were calculated with the tool *vegan::diversity* within the *vegan* v. 2.6‐4 R package [40]. The Bray‐Curtis dissimilarity matrix was built using the *vegan::vegdist* tool, which is also included in the *vegan* R package. Using the Bray‐Curtis dissimilarity matrix, principal coordinate analysis (PCoA) was performed using the *stats::cmdscale* tool in the base *Stats* R package. Permutational multivariate analysis of variance (PERMANOVA) was also performed with the Bray‐Curtis dissimilarity matrix, applying 10,000 permutations using the *vegan::adonis2* tool (*vegan* R package).

## 3 Results

### 3.1 Plant Growth and Nodulation

**Figure 1:**
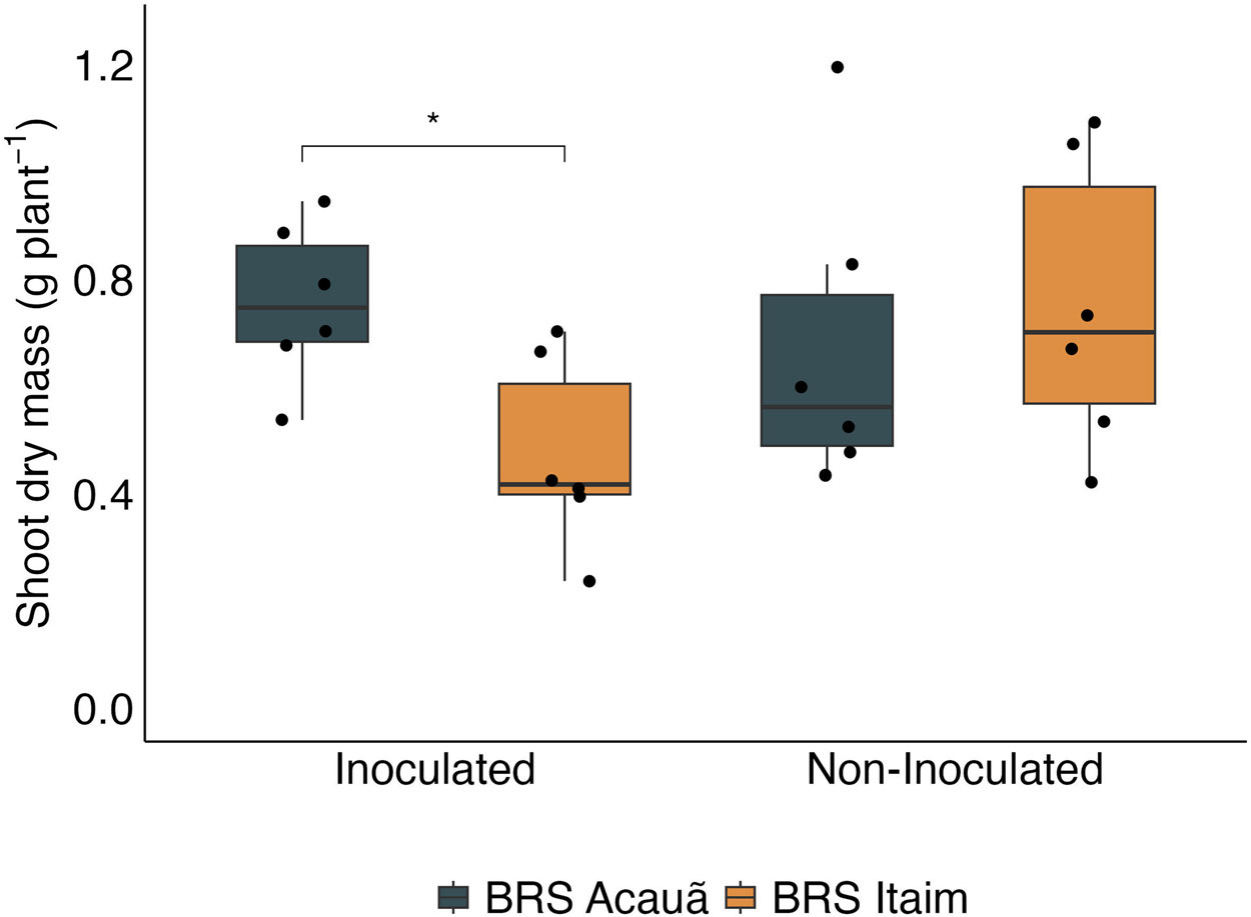
Interaction of plant genotype (BRS Acauã and BRS Itaim) and inoculation of *Bacillus* sp. ESA 402 (inoculated or non‐inoculated) cowpea' shoot dry mass (*n* = 6). * = *p* < 0.05.

**Figure 2:**
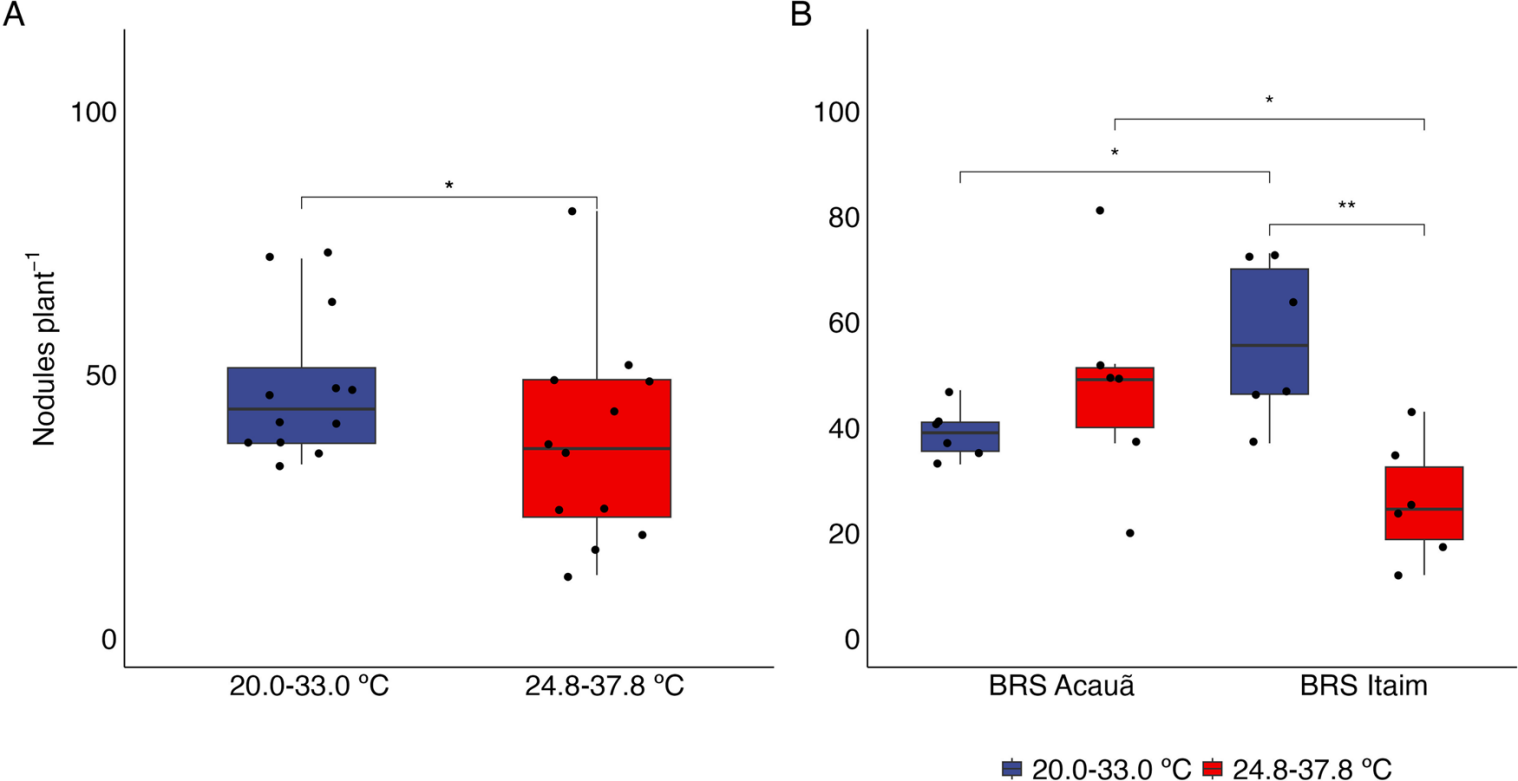
Influence of the air temperature as a single factor (*n* = 12) (A) or the interaction between the temperature and the cowpea genotype (BRS Acauã and BRS Itaim) (B, *n* = 6) on the number of nodules per plant. * = *p* < 0.05, ** = *p* < 0.01.

The interaction of the three factors (plant genotype, temperature, and inoculation of *Bacillus* sp. ESA 402) did not affect cowpea growth and nodulation (Table S3). However, the plant genotype and ESA 402 inoculation interaction affected the variable shoot dry mass (*p* < 0.05). Although a significant F‐test value was detected, there was no difference in the interactive effect of plant genotype and inoculation in the two‐tailed *t*‐test (Figure 1). Temperature influenced cowpea nodulation since the plants grown at 20.0–30.0° C showed higher nodulation than those grown at 24.8–37.8° C at *p* < 0.05 (Figure 2A). BRS Acauã had more nodules per plant than BRS Itaim under the higher temperature regime (*p* < 0.05). However, the opposite was observed in the lower temperature regime, where BRS Itaim nodulated more than BRS Acauã (*p* < 0.01). For the genotype BRS Itaim, increasing the air temperature drastically reduced nodulation, while BRS Acauã showed no differences in the number of nodules per plant when comparing both temperature regimes (Figure 2B).

### 3.2 Rhizobia Isolation, Molecular Authentication, and Genetic Variability

**Figure 3:**
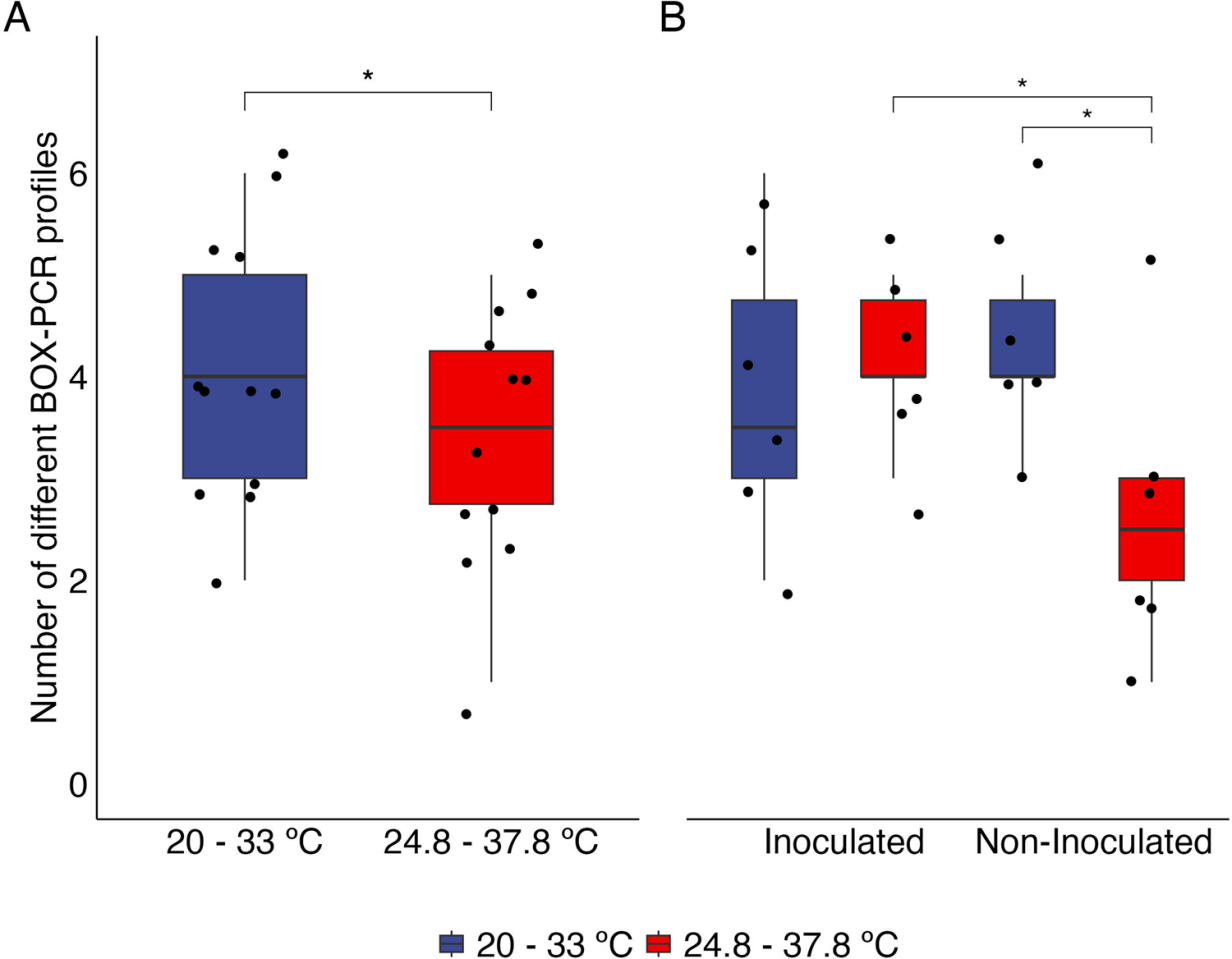
Different BOX‐PCR profiles by air temperature (A) and by the interaction between the air temperature (20.0–33.0°C or 24.8–37.8°C) and the genotype (BRS Acauã and BRS Itaim) (B), *n* = 6). * = *p* < 0.05.

We obtained 230 isolates from cowpea nodules, which showed typical bradyrhizobia culture characteristics grown in the YMA medium. We were able to amplify *nodC* and *nifH* from 186 (80.9 %) of those isolates. The isolates containing *nodC* and *nifH* were fingerprinted by BOX‐PCR and clustered in 45 groups with 100% similarity. Among these clusters, 18 were formed by a single bacterial strain. Otherwise, 27 clusters were formed by two or more bacteria. Considering the total number of different bacteria per treatment, we found differences influenced by the air temperature of incubation and the interaction between plant genotype and inoculation or not with ESA 402 (Table S4). The lower incubation temperatures favored the occurrence of total bacterial profiles compared to the higher incubation air temperatures (*p* < 0.05) (Figure 3A). The higher temperature reduced the amount of BOX‐PCR profiles of the non‐inoculated cowpea plants (*p* < 0.05), while no difference between the temperature regimes could be observed in the ESA 402 inoculated plants. Moreover, when comparing the number of genetic profiles for the plants under the higher temperature regime, the plants that were not inoculated with ESA 402 showed fewer bradyrhizobia genetic profiles compared to the inoculated plants (*p* < 0.05) (Figure 3B).

**Figure 4:**
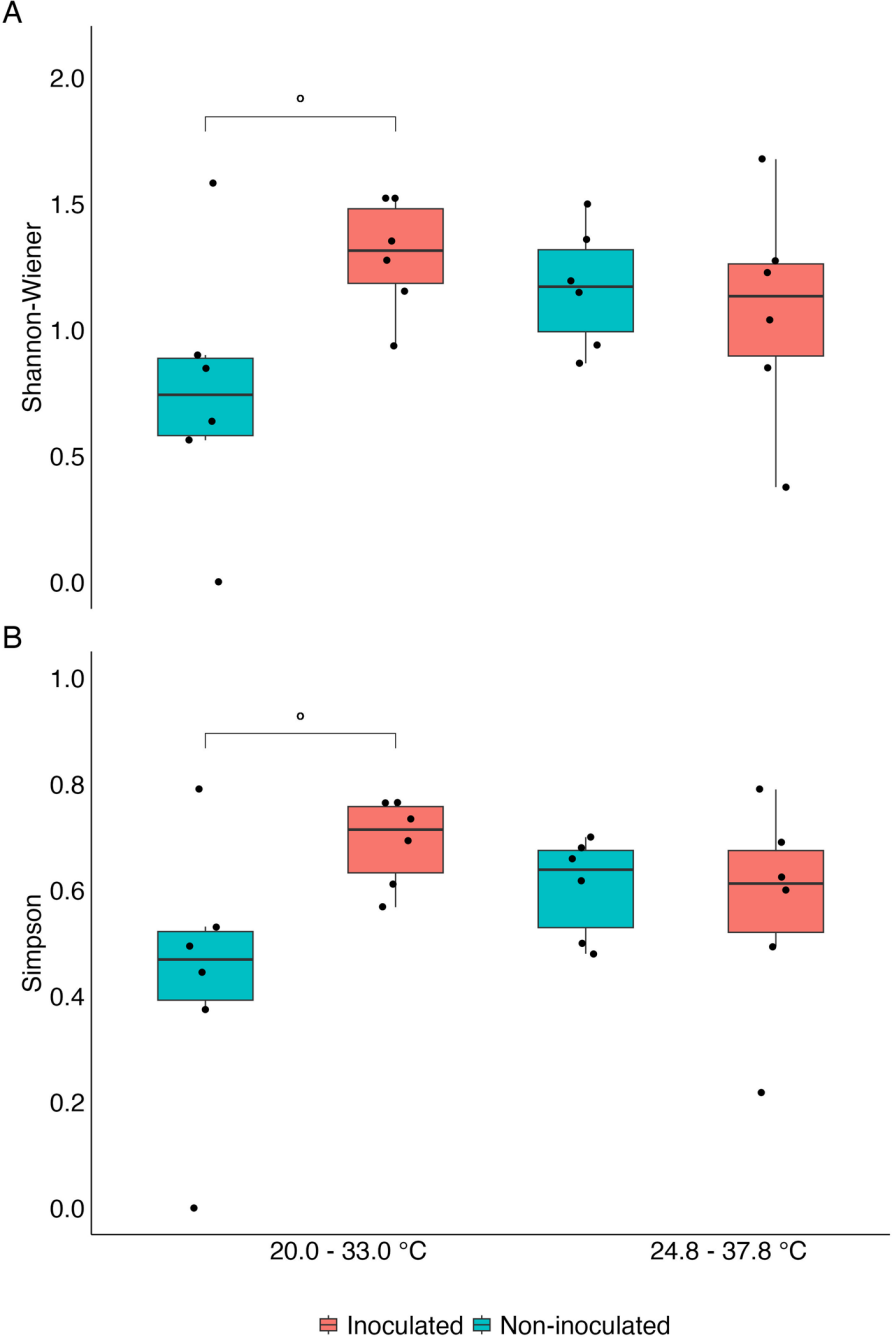
Shannon‐Wiener (A) and Simpson (B) indexes based on the occurrence and abundance of 45 different BOX‐PCR genetic profiles. Indexes calculated by the interaction of the inoculation of *Bacillus* sp. ESA 402 (inoculated or non‐inoculated) and the interaction between the air temperature (20.0–33.0°C or 24.8–37.8°C) (*n* = 6). ° = *p* < 0.1.

The alpha diversity Shannon‐Wiener and Simpson indexes showed no significance in single factors and triple interaction. However, the double interaction between the inoculation or not with ESA 402 and the temperature regime was slightly significant at *p* < 0.1 (Table S5). Comparing the levels of the combined interaction, we observed that, for both indexes, the inoculation of the *Bacillus* sp. ESA 402 strain improved the bacterial diversity based on the total number of clusters (Shannon) but reduced the diversity based on the relative abundance of the different isolates (Simpson) (Figure 4).

**Figure 5:**
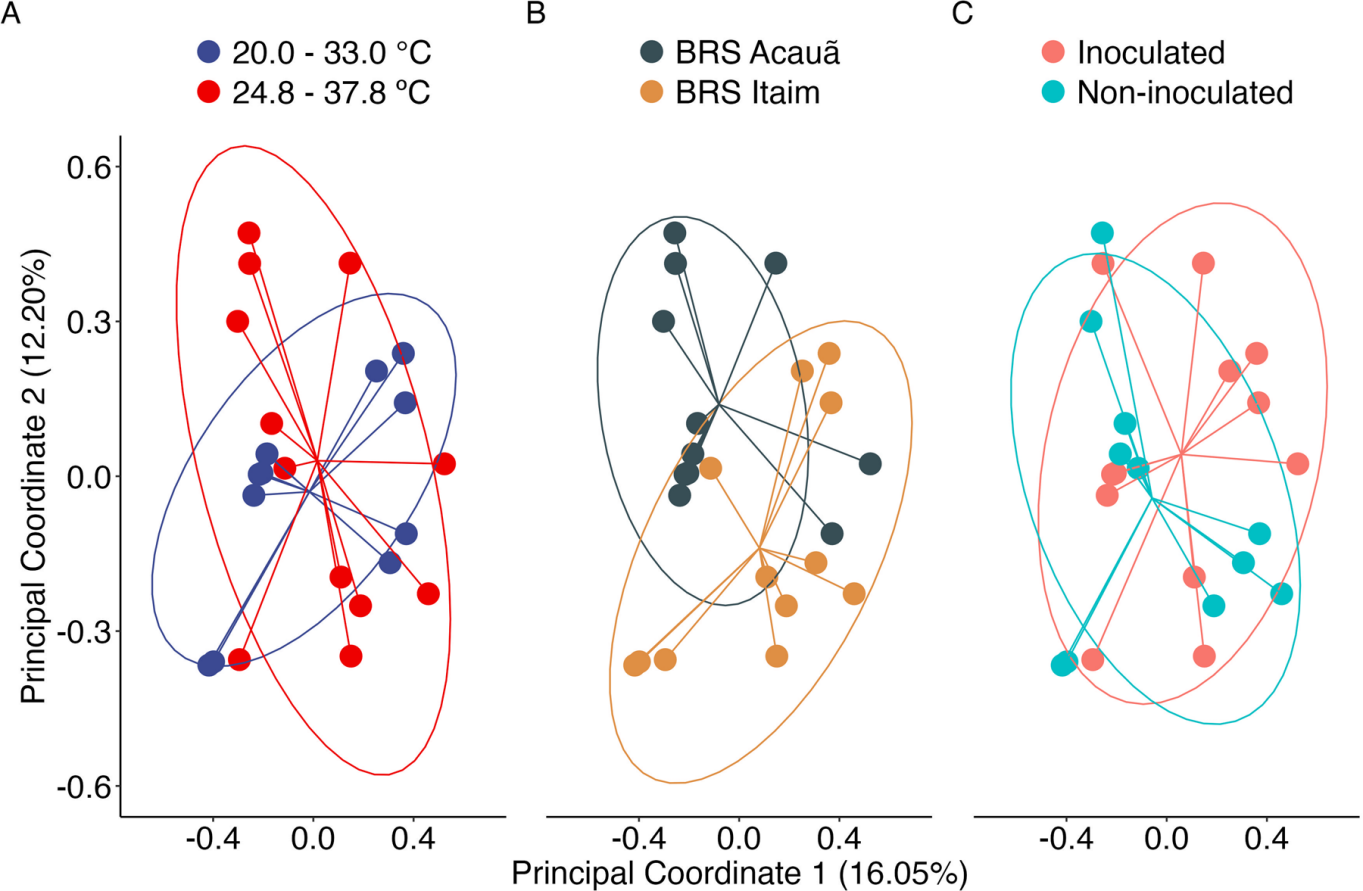
Principal coordinates analysis (PCoA) by bacterial beta diversity among different factors and their interactions determined through PERMANOVA based on the Bray–Curtis distance matrix.

A principal coordinates analysis (PCoA) conducted with the Bray‐Curtis dissimilarity matrix showed that PCo1 and PCo2 explained 28.25% of the total variance observed (Figure 5). The permutational multivariate analysis of variance (PERMANOVA) analyzing the Bray‐Curtis dissimilarity matrix showed significant differences in all single factors and the triple interaction, as well as for the double interactions between plant genotype and ESA 402 inoculation, in addition to the plant genotype and air temperature (Table 1), indicating the multifactorial influence on bradyrhizobia nodulation in different cowpea genotypes, *Bacillus* inoculation, and air temperature incubation.

**Table 1:** Permutational multivariate analysis of variance (PERMANOVA) summary table for the Bray–Curtis dissimilarity matrix from the distribution of the 45 different Box‐PCR profiles of 186 bacteria from root nodules of cowpea (*Vigna unguiculata* L. Walp.).

	Degrees of freedom	Sum of squares	R^2^	F	*p* value
Genotype	1	0.664	0.064	2.036	0.006
Inoculation (ESA 402)	1	0.887	0.085	2.720	0.000
Temperature	1	0.605	0.058	1.855	0.015
Genotype*Inoculation	1	0.928	0.089	2.845	0.000
Genotype*Temperature	1	0.678	0.065	2.078	0.005
Inoculation*Temperature	1	0.426	0.041	1.305	0.168
Genotype*Inoculation*Temperature	1	0.979	0.094	3.001	0.000
Residuals (error)	16	5.219	0.503		
Total	23	10.386	1.000		

*Note:* PERMANOVA with 10,000 permutations.

### 3.3 Phylogenetic Analyses of the 16S rRNA Gene Sequences and Housekeeping Gene Sequences

**Figure 6:**
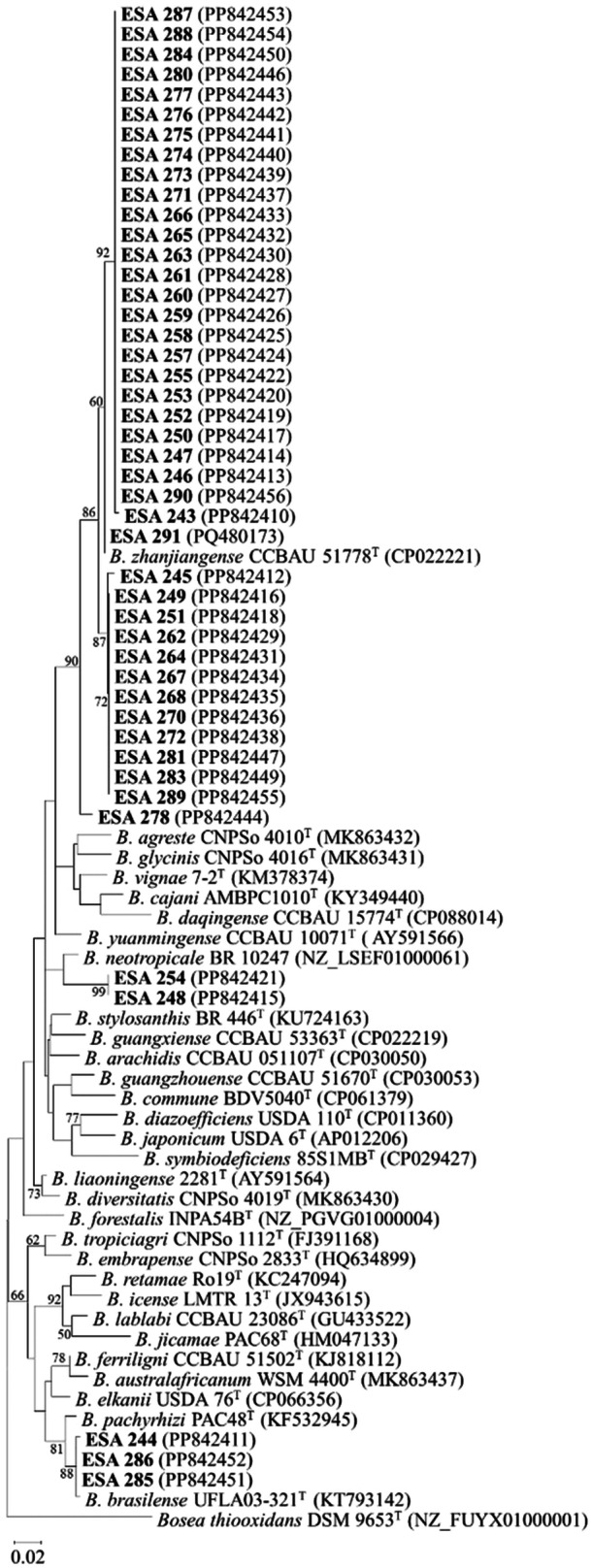
Maximum‐likelihood phylogenetic tree of the 16S rRNA gene sequences of 45 *Bradyrhizobium* strains from *Vigna unguiculata* root‐nodules plus 30 type strains (917 nucleotides). The numbers in the branches are the bootstrap values > 50% (1000 replications). *Bosea thiooxidans* DSM 9653 ^T^ was included as an outgroup. “ESA” strains are those from the current study and are shown in boldface with their GenBank accession number in parenthesis. Jukes‐Cantor model was used for phylogenetic reconstruction.

The 16S rRNA phylogeny showed that all 45 strains were classified as *Bradyrhizobium* spp. Forty‐two strains are clustered in the *B. japonicum* superclade, and three strains are clustered in *B. elkanii* superclade (Figure 6). The 42 superclade *B. japonicum* strains formed a consistent cluster with *B. zhanjiangense* CCBAU 51778 ^T^ with 90% bootstrap support. The *B. elkanii* superclade strains (ESA 244, ESA 285, and ESA 286) clustered with different type strains, including *B. elkanii* USDA 76 and *B. neotropicale* BR 10247, however, with a low bootstrap support (< 50%).

**Figure 7:**
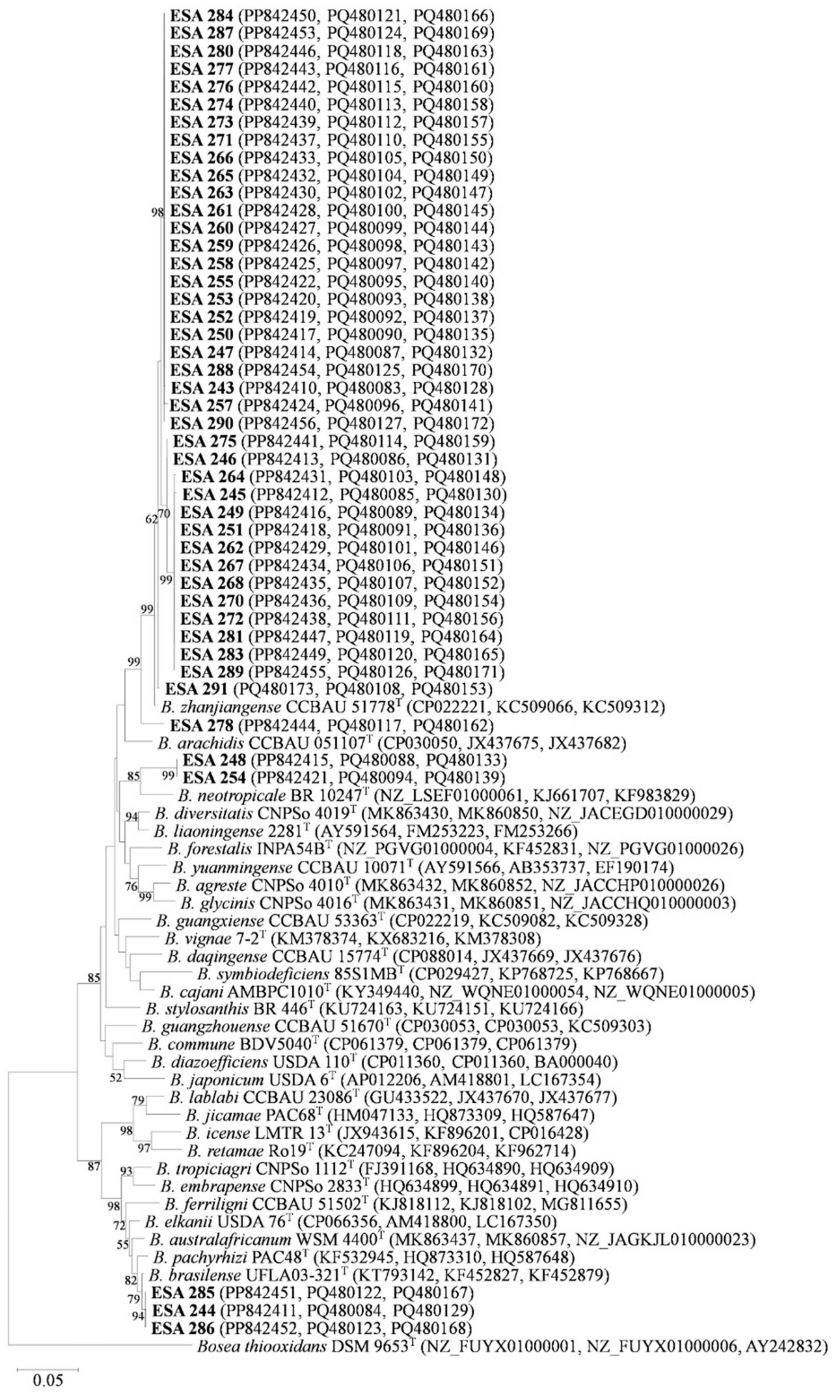
Maximum‐likelihood phylogenetic tree based on the concatenated sequence data for the genes (*recA*, *gyrB*, and *rpoB*) of 45 *Bradyrhizobium* strains and 30 type strains (1171 nucleotides) isolated from root nodules of *Vigna unguiculata*. The numbers in the branches are the bootstrap values > 50% (1000 replications). *Bosea thiooxidans* DSM 9653 ^T^ was included as an outgroup. “ESA” strains are those from the current study and are shown in boldface with their GenBank accession number in parenthesis. Jukes‐Cantor model was used for phylogenetic reconstruction.

Nucleotide sequences from the *recA*, *gyrB*, and *rpoB* housekeeping genes of the 45 strains were used to evaluate their phylogenetic relationships. The phylogenies of single housekeeping genes *recA* (372 bp), *gyrB* (440 bp), and *rpoB* (335 bp) were able to differentiate the *Bradyrhizobium* strains in at least four different clusters (Figures S1–S3). The overall phylogeny achieved from each housekeeping gene sequence analysis was congruent. To avoid possible discrepancies caused by events of recombination at a single locus, a Multilocus Sequence Analysis (MLSA) was performed with the partial sequences of the housekeeping genes *recA*+*gyrB*+*rpoB* (1171 bp) (Figure 7). Based on the MLSA tree, the 45 strains of this study were not grouped with any of the type strains of *Bradyrhizobium*.

Forty‐two strains were placed in *B. japonicum* superclade, while three strains remained clustered within *B. elkanii* superclade, in agreement with the 16S rRNA phylogeny. Forty strains formed a large cluster with *B. zhanjiangense* CCBAU 51778 ^T^ with 99% bootstrap support. At least two smaller clusters were formed: one with 15 strains (70% bootstrap support) and another with 24 strains (98% bootstrap support). The strains ESA 278 and ESA 291 were not grouped with any of the other strains in this study, nor with *Bradyrhizobium* type strains. Two other *B. japonicum* superclade strains (ESA 248 and ESA 254) also formed a cluster with 99% bootstrap support, showing a closer relationship with *B. neotropicale* BR 10247 ^T^. The *B. elkanii* superclade strains ESA 244, ESA 285, and ESA 286 clustered with *B. brasilense* UFLA03‐321^T^ with a 94% bootstrap support.

## 4 Discussion

Multiple factors, such as air temperature and the use of microbial inoculants, affect cowpea growth and development. Temperature affects various aspects of cowpea biology, including flower development and abortion, pollen viability, and plant growth [12, 14, 29]. In addition to the existing literature reports on how temperature influences cowpea's aboveground development, this study highlights the significance of air temperature for belowground cowpea development and its interaction with soil bacteria, which affects nodulation with the established autochthonous rhizobial community. A plethora of native bradyrhizobia from the Brazilian drylands can abundantly nodulate and fix N in association with cowpeas [6, 41, 42], but nodulation and N fixation vary among plant genotypes [6, 7, 41, 43, 44]. Elevated temperatures, can also impact the symbiosis of the native bradyrhizobial community, as we observed that BRS Itaim had reduced nodulation under the high‐temperature regime that did not affect the nodulation of BRS Acauã. This result is important for improved cowpea‐bradyrhizobia symbiosis under future climatic conditions.

In addition to nodulation, increased air temperature reduced the number of rhizobial strains in cowpea nodules. Other environmental stresses, including drought or salinity, have been shown to reduce the total diversity of chickpea (*Cicer arietinum*) nodulating rhizobia [45], for example. However, the role of increased air temperature on the nodulation of legumes has not been consistently reported. The PGPB *Bacillus* sp. ESA 402 strain was isolated from sorghum plants under field growth conditions in the Brazilian drylands [24]. This bacterium also induced plant‐drought tolerance when inoculated into sorghum [25] and sesame [26, 27]. In the present study, there was a decrease in the total genetic profiles of bradyrhizobia when subjected to high air temperature in the absence of *Bacillus* sp. ESA 402 indicates that this plant growth‐promoting bacterium modulates the diversity of cowpea nodulating rhizobia, under heat‐stress conditions.

Despite the previous evidence that this bacterium is an effective tool for improving drought tolerance in nonlegume crops, this is the first study evaluating its efficiency under high‐temperature stress. Our findings indicate that, in addition to enhancing plant growth and resilience under water deficit, ESA 402 can also improve legume rhizobia diversity under heat stress, suggesting that the action of the bacteria when inoculated into the sorghum and sesame plants subjected to drought conditions (as well as on cowpeas under heat stress) could be related to regulating the rhizospheric microbial community. Additionally, these results indicate that using ESA 402 as an inoculant reduces the negative impact of community changes in cowpea nodule bradyrhizobial composition, possibly minimizing the adverse effects of heat on cowpea biological nitrogen fixation.

The alpha‐diversity assessment showed no differences among the sole factors and a weak difference (*p* < 0.1) regarding the interaction between the factors ESA 402 inoculation and the air temperature. Differences between the cowpea rhizobia communities assessed through alpha‐diversity metrics, such as Shannon‐Wiener and Simpson indexes, were already shown when comparing different soil samples (locations) but not when biochar application treatments were evaluated within the same soil sample [46]. In the present study, as well, since the diversity of bacteria in a single soil was not influenced by the factors (treatments) that the plants and soil rhizobia were subjected to. Soil management, cover crop, and biogeography influence the alpha‐diversity of cowpea rhizobial communities [17, 47, 48]. However, detecting differences within the same sampling site is uncommon, even when different genotypes are grown or soil amendments are applied.

Increasing air temperature may modify the composition of soil microbial communities [49, 50] and root exudation patterns [19, 51], thereby affecting plant‐microbe interactions. The PERMANOVA analysis of the Bray‐Curtis dissimilarity matrix captured this influence in our study. The lack of convergence between alpha‐diversity (within samples/treatments) and beta‐diversity analysis (among samples/treatments) shows that the changes in genetic clusters themselves (alpha) do not reflect the changes observed when the samples/treatments are compared (beta), what supports the occurrence of different genetic clusters per treatments, even with the same alpha‐diversity metrics. In this case, the point analysis of the treatments could not reflect the changes observed among the treatments applied, reflecting the interaction of different levels of cowpea genotypes, air temperature, or presence/absence of PGPB *Bacillus* sp. ESA 402. By the way, the inoculation of ESA 402 increased the number of different genetic profiles nodulating cowpeas under higher temperatures, minimizing the effects of heat stress.

The future climate, characterized by increased air temperatures, will be accompanied by various other climatic changes, including reduced water availability. In this challenging environment, climate conditions will influence rhizospheric microbiomes [49, 50] and their effects on plants and their root exudation patterns [19, 51]. The development of adaptation technologies, such as microbial inoculants that diminish the impact of harsh climatic conditions on plants, is an urgent theme. In this context, *Bacillus* sp. ESA 402 stands out as a candidate for the development of commercial inoculants since this bacterium can be used as a tool to endure crops under drought conditions, as already reported [25, 26, 27], and also work as a homeostasis inducing strain under heat conditions maintaining the natural diversity of cowpea nodulating bradyrhizobia, the first step to retain the cowpea's nitrogen fixation ability. Further studies must be conducted to estimate cowpea's nitrogen fixation under the conditions assessed in the present study.

The bacterial identification through 16S rRNA, *recA*, *gyrB*, and *rpoB* gene sequences indicated that all bacterial strains belonged to the *Bradyrhizobium* genus, corroborating the culture characteristics of the culture collection in the YMA medium. The classification of 43 out of 45 bacterial isolates as members of *B. japonicum* supercluster agrees with previous results of cowpea's *Bradyrhizobium* diversity studies in agricultural soils of the Brazilian drylands [17, 46, 47] supporting the hypothesis that cowpeas nodulate “preferentially” with those strains belonging to the same cluster [52]. Most of the *B. japonicum* superclade strains were related to *B. zhanjiangense* CCBAU 51778 ^T^ in the trees 16S rRNA and *recA*+*gyrB*+*rpoB* concatenated sequences, a species widespread in agricultural soils of drylands in Brazil [17, 46, 53]. However, all the 45 strains of this study were not grouped with any *Bradyrhizobium*‐type strains in the trees. Thus, the phylogenetic analysis of housekeeping genes revealed at least four groups with the possible presence of still non‐described species.

It should be noted that for the 16S rRNA gene tree the strains ESA 244, ESA 285, and ESA 286 clustered into superclade *B. elkanii* with different type strains, including *B. elkanii* USDA 76 ^T^ and *B. neotropicale* BR 10247 ^T^, with low bootstrap support (< 50%). In the single *recA*, *gyrB*, and *rpoB* housekeeping genes and concatenated (*recA *+ *gyrB *+ *rpoB*) trees, ESA 248 and ESA 254 strains formed a cluster with 99% bootstrap support, showing a closer relation with *B. neotropicale* BR 10247 ^T^. These two strains for the 16S rRNA gene and all housekeeping genes were in the same superclade (*B. japonicum*). On the other hand, *B. neotropicale* BR 10247 ^T^ for the 16S rRNA gene was placed inside *B. elkanii* superclade, and for the housekeeping genes (*recA*, *gyrB*, and *rpoB*) was in the superclade *B. japonicum* one. In the case of *B. neotropicale* BR 10247 ^T^, clusters with different superclades when analyzing between 16S rRNA and housekeeping genes, suggesting a phylogenetic incongruence that must be addressed with further genetic analysis of this type strain.

The low diversity observed at the species level of the culture collection does not reflect the high diversity of our collection at the sub‐specific level, revealed by the BOX‐PCR profiles. These findings indicate the multifactorial influences of air temperature, PGPB inoculation, and plant genotype at the sub‐specific levels (strain level) rather than the species level in cowpea‐bradyrhizobia and the association under the conditions assessed.

Future climate conditions depend on the air temperature increasing to the worst scenario predicted by the IPCC, which will alter the diversity of cowpea bradyrhizobia at strains rather than species levels. *Bacillus* sp. inoculation can diminish the impact of high air temperatures on the cowpea‐bradyrhizobia association. ESA 402 may be a potential PGPB inoculant for reducing abiotic stresses that impact essential crops, such as cowpeas.

## Author Contributions

**Crislaine Soares Oliveira:** data curation, investigation, validation, writing – review and editing. **Juliane Rafaele Alves Barros:** investigation, writing – review and editing. **Viviane Siqueira Lima Silva:** investigation, writing – review and editing. **Paula Rose Almeida Ribeiro:** conceptualization, data curation, formal analysis, methodology, supervision, visualization, writing – review and editing, software. **Francislene Angelotti:** methodology, writing – review and editing, conceptualization, project administration, resources, supervision. **Paulo Ivan Fernandes‐Júnior:** conceptualization, formal analysis, methodology, funding acquisition, writing – original draft, writing – review and editing, project administration, resources, supervision, visualization, validation, software.

## Supporting information

069SupportingInformation.

069figS1.

069figS2.

069figS3.

## Data Availability

The data supporting the findings of this study are available on request from the corresponding author.
