# Supplementary material for: High Temperatures and Bacillus Inoculation Affect the Diversity of Bradyrhizobia in Cowpea Root Nodules
Source: J Basic Microbiol. 2025 May 20;65(9):e70058. doi: 10.1002/jobm.70058 (PMC12406091; doi:10.1002/jobm.70058)
Supplement: Supplementary file 1 — 069SupportingInformation. [file JOBM-65-e70058-s004.docx]

**Supporting Information**

**Legends of figures**

**Figure S1** Maximum-likelihood phylogenetic tree of the *recA* gene sequences of 45 *Bradyrhizobium* strains and 30 type strains (372 nucleotides) isolated from root nodules of *Vigna unguiculata*. The numbers in the branches are the bootstrap values > 50% (1000 replications). *Bosea thiooxidans* DSM 9653^T^ was included as an outgroup. “ESA” strains are those from the current study and are shown in boldface with their GenBank accession number in parenthesis. Jukes-Cantor model was used for phylogenetic reconstruction.

**Figure S2** Maximum-likelihood phylogenetic tree of the *gyrB* gene sequences of 45 *Bradyrhizobium* strains and 30 type strains (440 nucleotides) isolated from root nodules of *Vigna unguiculata*. The numbers in the branches are the bootstrap values > 50% (1000 replications). *Bosea thiooxidans* DSM 9653^T^ was included as an outgroup. “ESA” strains are those from the current study and are shown in boldface with their GenBank accession number in parenthesis. Jukes-Cantor model was used for phylogenetic reconstruction.

**Figure S3** Maximum-likelihood phylogenetic tree of the *rpoB* gene sequences of 45 *Bradyrhizobium* strains and 30 type strains (335 nucleotides) isolated from root nodules of *Vigna unguiculata*. The numbers in the branches are the bootstrap values > 50% (1000 replications). *Bosea thiooxidans* DSM 9653^T^ was included as an outgroup. “ESA” strains are those from the current study and are shown in boldface with their GenBank accession number in parenthesis. Jukes-Cantor model was used for phylogenetic reconstruction.

**Table S1** Chemical and physical characteristics of the experiment's topsoil samples [according to the methods recommended by Teixeira et al. (2017)].

| Depth | EC | pH | P | K+ | Na+ | Ca²+ | Mg²+ | Al³+ | H+Al | SB | CEC |
| --- | --- | --- | --- | --- | --- | --- | --- | --- | --- | --- | --- |
| cm | dS m | H2O | mg dm-³ | ---------------------------cmolc_c_ dm^-3^ ------------------ | | | | | | | |
| 0-0.2 | 1.81 | 6.29 | 1.1 | 0.56 | 0.07 | 2.74 | 1.11 | 0.0 | 0.59 | 6.9 | 5.07 |
| Depth | | V | | Cu | | Fe | | Mn | | Zn | |
| m | | % | | --------------------------mg dm^-3^------------------------------------ | | | | | | | |
| 0-0.2 | | 88.44 | | 1.04 | | 57.3 | | 36.1 | | 0.4 | |

**Table S2** Oligonucleotide primers used and PCR cycling conditions.

| Primer | Sequence (5'- 3') | Target gene (position) | PCR cycling | Reference |
| --- | --- | --- | --- | --- |
| TSrecA F  TSrecA R | CAACTGCMYTGCGTATCGTCGAAGG  CGGATCTGGATGAAGATCACCATG | *recA* (8-32)  *recA* (620-594) | 5 min 94 °C, 35X (45s 94 °C, 30s 58 °C, 1.30 min 72 °C) and 1X 7 min 72 °C. | Stepkowski et al. (2005) |
|  |  |  |  |  |
| gyrB343 F  gyrB1043 R | TTCGACCGAAAYTCCTACAGG  AGCTTGTCCTTSGTCTGCG | *gyrB* (343-364)  *gyrB* (1061-1043) | 5 min 95 °C, 35X (1 min 94 °C, 1 min 58 °C, 1 min 72 °C) and 1X 5 min 72 °C. | Martens et al. (2008) |
|  |  |  |  |  |
| rpoB456 F  rpoB1364 R | ATCGTYTCGCAGATGGACCG  TCGATGTCGTCGATYTCGCC | *rpoB* (456-477)  *rpoB* (1364-1385) | 5 min 95 °C, 3X (2 min 94 °C, 2 min 58 °C, 1 min 72 °C), 30X (30s 94 °C, 1 min 58 °C, 1 min 72 °C) and 5 min 72 °C. | Vinuesa et al. (2008) |
|  |  |  |  |  |
| 16S Y1 F  16S Y3 R | TGGCTCAGAACGAACGCTGGCGGC  ACGAGCTGACGACAGCCATGCAGCACCT | 16S rRNA (20-42)  16S rRNA (1500-1522) | 4 min 94 °C, 35X (1 min 94 °C, 45s 60 °C, 2 min 72 °C) and 1X 5 min 72 °C. | Young et al. (1991) |
|  |  |  |  |  |

**Table S3**  Summary of the analysis of variance (ANOVA) tables for the variables shoot and root dry mass and the number of nodules per plant for the cowpeas growth experiment by the factors Genotype, ESA 402 inoculation, and two temperature regimes incubation in addition to their double and triple interaction (n = 3).

|  | Degrees of freedom | Mean Square | F value | *p*-value |
| --- | --- | --- | --- | --- |
|  | Shoot dry mass | | | |
| Genotype | 1 | 0.15 | 1.90 | 0.19 |
| Inoculation (ESA 402) | 1 | 0.10 | 1.24 | 0.28 |
| Temperature | 1 | 0.18 | 2.19 | 0.16 |
| Genotype*Inoculation | 1 | 0.40 | 4.95 | 0.04 |
| Genotype*Temperature | 1 | 0.10 | 1.23 | 0.28 |
| Inoculation*Temperature | 1 | 0.20 | 2.49 | 0.13 |
| Genotype*Inoculation*Temperature | 1 | 0.00 | 0.02 | 0.90 |
| Residuals (Error) | 16 | 0.08 |  |  |
|  | Root dry mass | | | |
| Genotype | 1 | 0.94 | 2.67 | 0.12 |
| Inoculation (ESA 402) | 1 | 0.30 | 0.86 | 0.37 |
| Temperature | 1 | 0.00 | 0.01 | 0.92 |
| Genotype*Inoculation | 1 | 0.10 | 0.29 | 0.60 |
| Genotype*Temperature | 1 | 0.01 | 0.03 | 0.86 |
| Inoculation*Temperature | 1 | 0.08 | 0.22 | 0.65 |
| Genotype*Inoculation*Temperature | 1 | 0.10 | 0.30 | 0.59 |
| Residuals (Error) | 16 | 0.35 |  |  |
|  | Nodules per plant | | | |
| Genotype | 1 | 2.58 | 0.32 | 0.58 |
| Inoculation (ESA 402) | 1 | 2.05 | 0.26 | 0.62 |
| Temperature | 1 | 29.74 | 3.74 | 0.07 |
| Genotype*Inoculation | 1 | 0.04 | 0.01 | 0.94 |
| Genotype*Temperature | 1 | 77.00 | 9.68 | 0.01 |
| Inoculation*Temperature | 1 | 0.79 | 0.10 | 0.76 |
| Genotype*Inoculation*Temperature | 1 | 0.91 | 0.11 | 0.74 |
| Residuals (Error) | 16 | 7.95 |  |  |

**Table S4**  Summary of the analysis of variance (ANOVA) tables for the total number of bacterial isolates and the number of different box-PCR genetic profiles by the factors Genotype, ESA 402 inoculation, and two temperature regimes incubation in addition to their double and triple interaction (n = 3).

|  | Degrees of freedom | Mean Square | F value | *p*-value |
| --- | --- | --- | --- | --- |
|  | Total bacterial isolates | | | |
| Genotype | 1 | 90.09 | 0.57 | 0.46 |
| Inoculation (ESA 402) | 1 | 0.84 | 0.01 | 0.94 |
| Temperature | 1 | 720.51 | 4.55 | 0.05 |
| Genotype*Inoculation | 1 | 882.09 | 5.57 | 0.03 |
| Genotype* Temperature | 1 | 38.76 | 0.24 | 0.63 |
| Inoculation* Temperature | 1 | 404.26 | 2.55 | 0.13 |
| Genotype*Inoculation* Temperature | 1 | 0.84 | 0.01 | 0.94 |
| Residuals (Error) | 16 | 158.34 |  |  |
|  | Amount of different Box-PCR Clusters | | | |
| Genotype | 1 | 0.03 | 0.29 | 0.60 |
| Inoculation (ESA 402) | 1 | 0.14 | 1.25 | 0.28 |
| Temperature | 1 | 0.21 | 1.90 | 0.19 |
| Genotype*Inoculation | 1 | 0.14 | 1.27 | 0.28 |
| Genotype* Temperature | 1 | 0.10 | 0.90 | 0.36 |
| Inoculation* Temperature | 1 | 0.52 | 4.68 | 0.05 |
| Genotype*Inoculation* Temperature | 1 | 0.01 | 0.08 | 0.78 |
| Residuals (Error) | 16 | 0.11 |  |  |

**Table S5** Summary of the analysis of variance (ANOVA) tables for the Shannon-Wiener and Simpson indexes by the factors Genotype, ESA 402 inoculation, and two temperature regimes incubation in addition to their double and triple interaction (n = 3).

|  | Degrees of freedom | Mean Square | F value | *p*-value |
| --- | --- | --- | --- | --- |
|  | Shannon-Wiener | | | |
| Genotype | 1 | 0.06 | 0.42 | 0.53 |
| Inoculation (ESA 402) | 1 | 0.30 | 1.97 | 0.18 |
| Temperature | 1 | 0.06 | 0.37 | 0.55 |
| Genotype*Inoculation | 1 | 0.11 | 0.72 | 0.41 |
| Genotype*Temperature | 1 | 0.23 | 1.50 | 0.24 |
| Inoculation*Temperature | 1 | 0.60 | 3.99 | 0.06 |
| Genotype*Inoculation*Temperature | 1 | 0.03 | 0.18 | 0.68 |
| Residuals (Error) | 16 | o.15 |  |  |
|  | Simpson | | | |
| Genotype | 1 | 0.01 | 0.27 | 0.61 |
| Inoculation (ESA 402) | 1 | 0.07 | 2.08 | 0.17 |
| Temperature | 1 | 0.00 | 0.10 | 0.76 |
| Genotype*Inoculation | 1 | 0.00 | 0.10 | 0.75 |
| Genotype*Temperature | 1 | 0.06 | 1.86 | 0.19 |
| Inoculation*Temperature | 1 | 0.12 | 3.73 | 0.07 |
| Genotype*Inoculation*Temperature | 1 | 0.00 | 0.05 | 0.83 |
| Residuals (Error) | 16 | 0.03 |  |  |
