# Supplementary figures and images for: High Temperatures and Bacillus Inoculation Affect the Diversity of Bradyrhizobia in Cowpea Root Nodules
Source: J Basic Microbiol. 2025 May 20;65(9):e70058. doi: 10.1002/jobm.70058 (PMC12406091; doi:10.1002/jobm.70058)

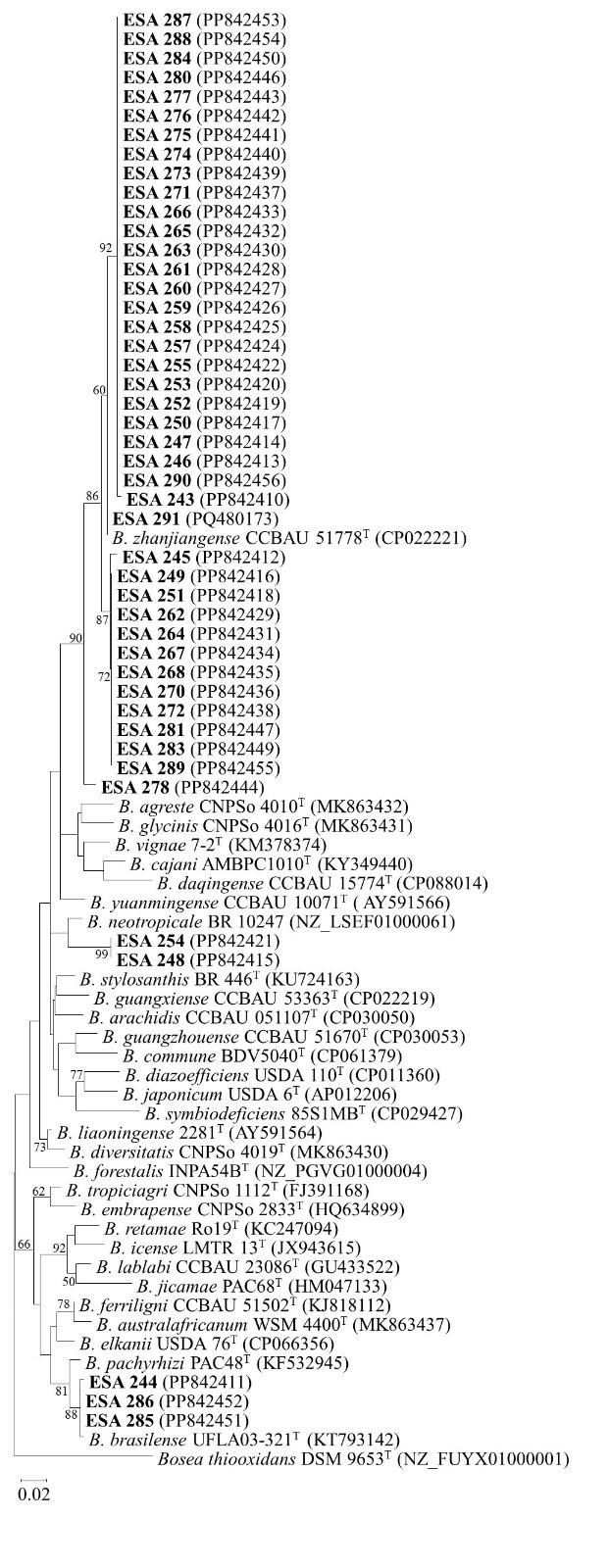

Supplement: Supplementary file 2 — 069figS1. [file JOBM-65-e70058-s001.jpeg]

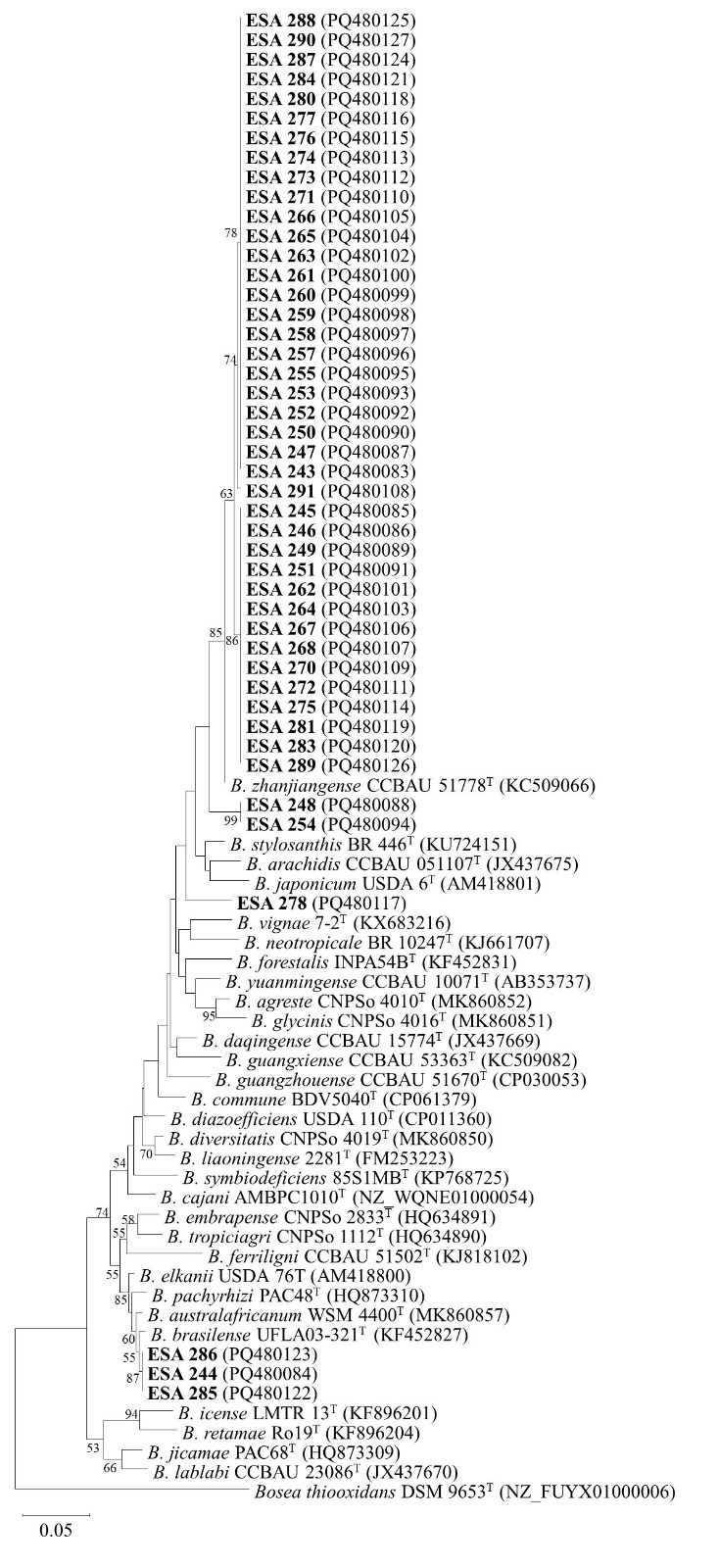

Supplement: Supplementary file 3 — 069figS2. [file JOBM-65-e70058-s002.jpg]

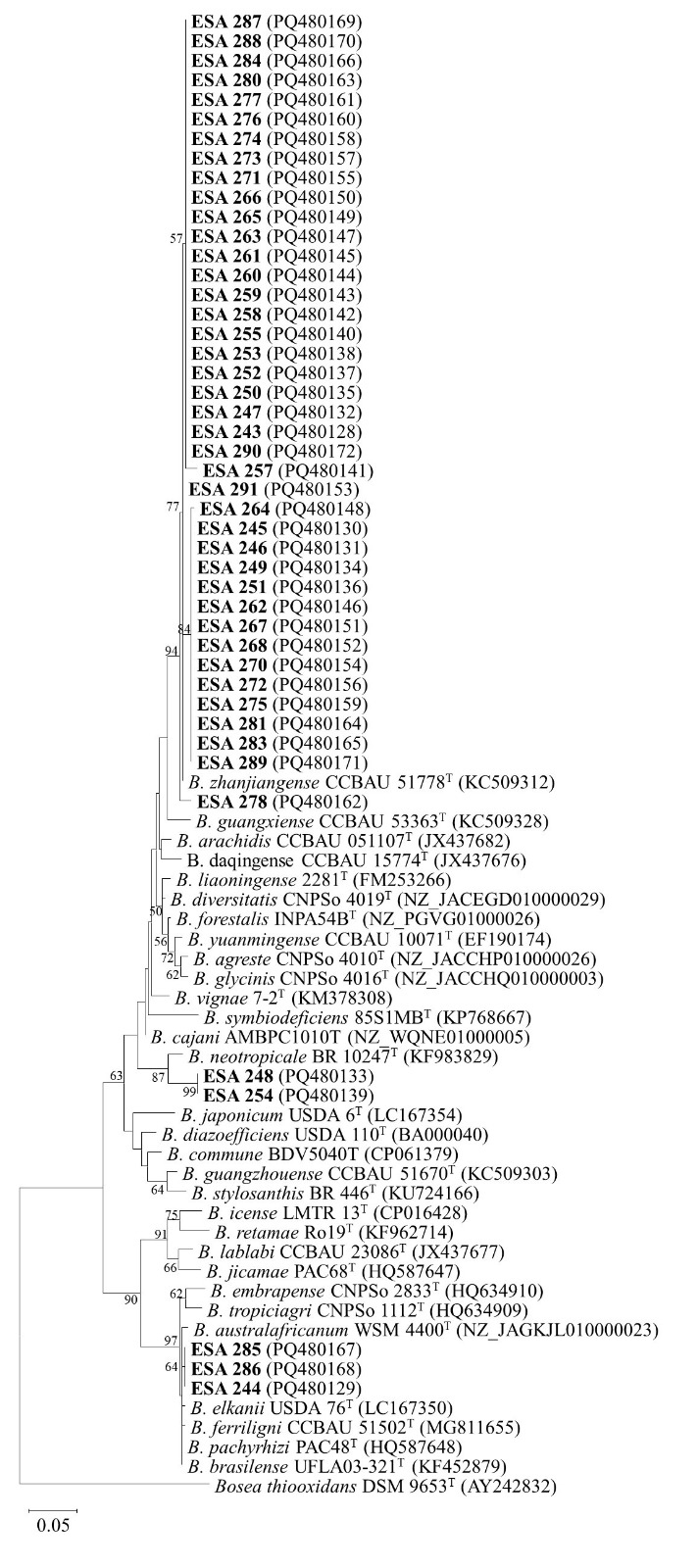

Supplement: Supplementary file 4 — 069figS3. [file JOBM-65-e70058-s003.jpeg]
